# Potential value of serum prealbumin and serum albumin in the identification of postoperative delirium in patients undergoing knee/hip replacement: an observational study and internal validation study

**DOI:** 10.3389/fneur.2024.1375383

**Published:** 2024-04-17

**Authors:** Bin Wang, Yan Xin, Xinhui Tang, Fei Wang, Shuhui Hua, Yunchao Yang, Shanling Xu, Hongyan Gong, Rui Dong, Yanan Lin, Chuan Li, Xu Lin, Yanlin Bi

**Affiliations:** ^1^Department of Anesthesiology, Qingdao Municipal Hospital, Qingdao, Shandong, China; ^2^Department of Endoscopy Center, Qingdao Municipal Hospital, Qingdao, Shandong, China; ^3^Department of Anesthesiology, Binzhou Medical University, Binzhou, Shandong, China; ^4^Department of Anesthesiology, Weifang Medical College, Weifang, Shandong, China

**Keywords:** serum albumin, perioperative neurocognitive disorders, biomarkers, neurodegeneration, serum prealbumin

## Abstract

**Background:**

Postoperative delirium (POD) is a common postoperative neurological complication that can lead to a variety of postoperative complications. At present, the pathogenesis of POD is unclear. This study aims to explore the relationship between serum prealbumin and serum albumin and POD and whether serum prealbumin and serum albumin influence POD through POD core pathology.

**Objective:**

We enrolled 500 Chinese Han patients between September 2020 to January 2023. We analyzed the risk and protective factors of POD using the multivariate logistic regression. We also assessed the predictive power of serum prealbumin, serum albumin, and both in combination with CSF POD biomarkers. We used Stata MP16.0. to examine whether the association between serum prealbumin and serum albumin and POD was mediated by CSF POD biomarkers, and conducted an internal validation study to verify the accuracy of the combination of serum prealbumin + serum albumin + CSF POD biomarkers for predicting POD. The model was visualized using ROC curve and decision curve analysis (DCA). DynNom and Shiny packages were used to create an online calculator. Ten patients who had POD occurring from February 2023 to October 2023 were selected for internal verification.

**Results:**

Finally, a total of 364 patients were included in our study. Levels of serum prealbumin, serum albumin in the POD group were lower than those in the NPOD group. The lever of serum prealbumin, serum albumin were protective factors for POD. The relationship between serum prealbumin, serum albumin and POD was partially mediated by T-tau (12.28%) and P-tau (20.61%). The model combining serum prealbumin and serum albumin and POD biomarkers exhibited a relatively better discriminatory ability to predict POD. DCA also showed that the combination of serum prealbumin and serum albumin and POD biomarkers brought high predictive benefits to patients. The dynamic online calculator can accurately predict the occurrence of POD in the internal validation study.

**Conclusion:**

Preoperative low serum prealbumin and serum albumin levels were the preoperative risk factors for POD, which is partly mediated by T-tau and P-tau. The model combining serum prealbumin and serum albumin and CSF POD biomarkers can accurately predict the occurrence of POD.

**Clinical trial registration:**

http://www.clinicaltrials.gov, identifier ChiCTR2000033439.

## Introduction

Postoperative delirium (POD) represents a severe complication following anesthesia and surgical procedures for patients undergoing surgical interventions (1, 2). POD is characterized by temporary or permanent cognitive decline, deterioration in language comprehension, and decreased social adaptation. It can lead to other complications such as Alzheimer's disease (AD), prolonged hospitalization, higher treatment costs (3) and even increased mortality. And POD mainly affects the elderly (>65 years) (4). Despite the prevalence and clinical significance of POD, its mechanisms are poorly understood and no reliable biomarkers have been reported in previous studies.

Amyloid beta (Aβ), including Aβ_40_ and Aβ_42_, is the major component of senile plaques in AD. Tau is a microtubule-associated protein in neurons, which is essential for the assembly and stability of microtubules (5). A recent study showed that pre-operative positive CSF Aβ and Tau might increase the risk of delirium following surgery (6). Another study showed pre-operative positive CSF Aβ, total tau (T-tau), and phosphorylated tau (P-tau) were strong independent predictors of postoperative delirium after elective arthroplasty in an elderly population without a prior diagnosis of dementia. Aβ and tau have been considered to play important roles in the pathogenesis of POD (7, 8).

Prealbumin, also known as transthyretin, has a half-life in plasma of 2 days, much shorter than that of albumin. Prealbumin is therefore more sensitive to changes in protein-energy status than albumin, and its concentration closely reflects recent dietary intake rather than overall nutritional status (9). Albumin is synthesized by liver parenchymal cells, and its half-life in plasma is about 15–19 days. Albumin is the most abundant protein in plasma, accounting for 40%−60% of the total plasma proteins (10). Although albumin synthesis rate is affected by dietary protein intake, it is mainly regulated by the plasma protein level. Albumin is not stored in the liver cells in large amounts, and it is present in trace amounts in all extracellular fluids. As a multi-functional protein, serum albumin (SA) has the functions of carrier, chaperone, antioxidant, amino acid source, osmoregulation and so on (11). As a carrier, it facilitates the stability and transport of hydrophobic and hydrophilic molecules, including free fatty acids, steroid hormones, drugs, and metal ions. As a chaperone, serum albumin binds to and protects other proteins. As an antioxidant, serum albumin is an important component of most antioxidant substances in plasma due to its free sulfhydryl. These functions make serum albumin a major player in general health, aging, and neurodegeneration. When the blood-brain barrier is damaged, albumin enters the brain tissue and can cause epilepsy and neurodegeneration (12). BBB dysfunction has been previously reported to play an important role in the pathogenesis of Alzheimer's disease (AD) (13, 14). Since POD shares similar neuropathological mechanisms with AD (15, 16), we hypothesize that serum albumin may be associated with POD.

Our study explored the associations of serum prealbumin and serum albumin with POD and CSF POD biomarkers, and investigated whether the effects of serum prealbumin and serum albumin on POD were mediated by POD core pathology [Aβ_42_, total-tau (T-tau), and phosphorylated-tau (P-tau)]. We also conducted an internal validation study to verify the accuracy of the combined use of serum prealbumin and serum albumin and CSF POD biomarkers for predicting POD occurrence. These analyses were conducted based on the Perioperative Neurocognitive Disorder and Biomarker Lifestyle (PNDABLE) study.

## Methods

### PNDABLE study

Aimed to investigate perioperative disorders associated to neurocognition, the PNDABLE research seeks to comprehend their pathogenesis, risk elements, and biomarkers, with a focus on identifying PND lifestyle risk factors among the Han people in the north of China. The study holds significant scientific and pragmatic utility for generating a standardized model for early prevention and diagnosis of these conditions in the non-demented Han individuals. Before the preoperative cerebrospinal fluid and blood extraction, all participants provided informed consent. The study was entered in the Chinese Clinical Trial Registry under the number ChiCTR2000033439 and has gained approval from the Ethics Committee of Qingdao Municipal Hospital.

### Participants

In the PNDABLE study, Han Chinese individuals between the ages of 50 and 90 years, weighing 50–80 kg, and classified as ASA I–II, who received unilateral total knee arthroplasty with epidural anesthesia at Qingdao Municipal Hospital in the period between September 2020 to January 2023 were included as participants. The study had specific exclusion criteria, which were as follows: (1) Participants with a preoperative MMSE score lower than 24 points. (2) Individuals with a history of drug abuse or psychotropic drug abuse, as well as those on extended periods of steroid or hormone drug usage. (3) Patients with pre-operative stage III–IV liver encephalopathy. (4) Those who had not long past complex surgeries. (5) Individuals with considerable disabilities to see or hear. (6) Patients who showed to coagulate irregularly prior to the operation. (7) Subjects with multiple sclerosis, head injury, infections in the central nervous system, neurodegenerative conditions excluding AD (such as epilepsy, Parkinson's Disease), or other significant neurological disorders. (8) Individuals diagnosed with substantial psychological disorders. (9) Patients suffering from critical systemic diseases (e.g., malignant cancer) that could potentially influence CSF or blood values of AD biomarkers, including Aβ and tau. (10) Subjects with family records of genetic conditions.

**Figure 1:**
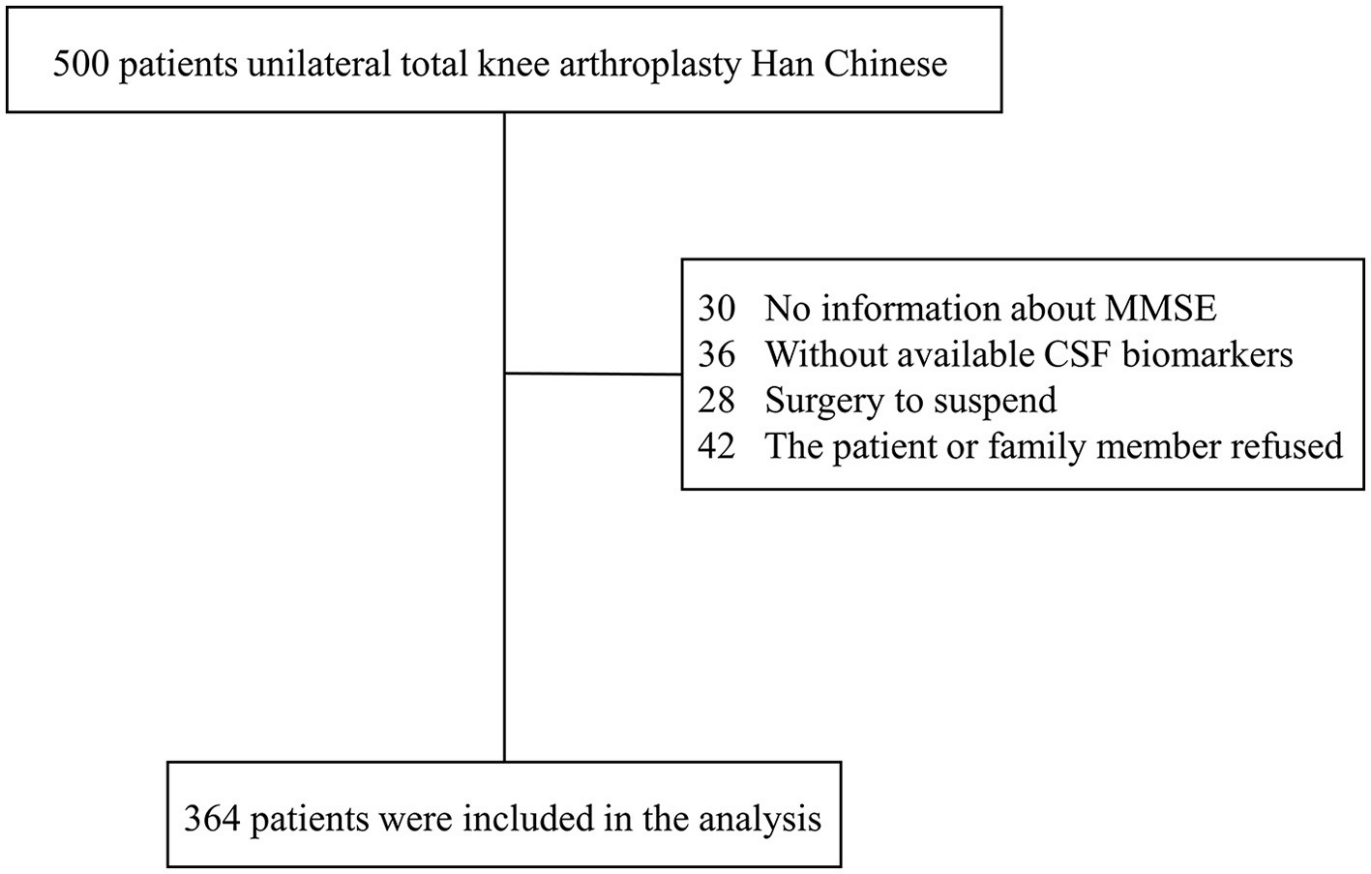
The flow diagram showed the selection of eligible patients and the enrollment process.

From the PNDABLE study, 500 participants with normal cognitive function contributed data on various covariates. These individuals were then classified into two groups: POD and NPOD, according to the occurrence or absence of postoperative cognitive dysfunction. The patient recruitment process is depicted in the flow chart of Figure 1.

To prepare for the procedure, all participants received specific instructions not to consume any food for 8 h and to refrain from drinking for 6 h prior to the scheduled time. Upon admission to the operating room, the participants received a combined spinal-epidural block, targeting the puncture location at the gap between lumbar 3–4 spinous processes (L3–4). Following successful puncturing, 2 ml of cerebrospinal fluid was taken from the subarachnoid space, and ~2–2.5 ml of ropivacaine (0.66%) was injected over a period of nearly 30 s. To sustain stable blood pressure during the surgical procedure, oxygen was administered through a mask at a flow rate of 5 L/min, with the goal of keeping the blood pressure in the range of +/– 20% of the baseline value. If the non-invasive blood pressure (NBP) fell below 90 mmHg (1 mmHg = 0.133 kPa) or was reduced higher than 20% of the baseline level, a 5 mg intravenous infusion of ephedrine was administered to the patient. Similarly, if the heart rate (HR) dropped below 50 beats per minute, a 0.5 mg intravenous infusion of atropine was given. For managing acute postoperative pain, an intravenous patient-controlled analgesia approach was employed. This included a mixture of butorphanol (0.1 mg/ml) and tropisetron (50 g/ml), which was diluted with normal saline until reaching a volume of 100 ml. Subsequent to the surgery, patients were transferred to the post-anesthesia care unit (PACU) for further observation and care.

Twenty-four hours before the surgery, comprehensive interviews were conducted with all patients to obtain their baseline data, encompassing age, gender, body mass index (BMI), and years of education. Moreover, relevant information, including medical history and comorbidities, was meticulously gathered from the patients' medical records. The preoperative cognitive status was assessed by neurologists. The task of conducting medical history collection, physical evaluations, and cognitive assessments related to dementia was undertaken by an anesthesiologist.

### Serum albumin and serum prealbumin measurements

The levels of serum albumin and serum prealbumin were measured from fasting venous blood within 24 h after admission. Analyses were performed by Olympus AU2700 automatic biochemical Analyzer.

### CSF POD biomarker measurements

Following the standard lumbar puncture, CSF samples were promptly processed within a 2-h window. Each sample underwent centrifugation at 2,000 × g during 10 min. The CSF samples underwent separation and were then stored in an enzyme-free EP (Eppendorf) tube (AXYGEN; PCR-02-C) at −80°C. These measures were taken under the international BIOMARKAPD project to facilitate their use in the following stages of this research project. CSF biomarkers of POD were quantified using ELISA with the aid of a microplate reader (Thermo Scientific Multiskan MK3). Specific ELISA kits, including Aβ_42_ (BioVendor, Ghent, Belgium Lot: No.296-64401), T-tau (BioVendor, Ghent, Belgium Lot: No. EK-H12242), and P-tau (BioVendor, Ghent, Belgium Lot: QY-PF9092), were employed for this purpose. To ensure accuracy and consistency, all ELISA measurements were carried out by senior technicians who strictly adhered to the manufacturer's instructions. These technicians were kept blinded to the clinical data during the measurement process. To minimize variability between batches, each sample and standard underwent duplicate measurements, and the mean of the duplicates was utilized for subsequent statistical analyses. Each antibody and plate used in the measurements originated from a single lot. Additionally, the within-batch coefficient of variation (CV) was maintained below 5%, while the inter-batch CV was kept below 15%, ensuring the reliability of the results.

### Neuropsychological tests

Neurologists performed a preoperative cognitive evaluation using the Mini-Mental State Examination (MMSE). Patients with an MMSE score below 24 points were excluded from the study.

Delirium assessment took place twice daily, between 9:00–10:00 am and 2:00–3:00 pm, starting from the first day until the seventh day after the operation (or prior to dismissal). An anesthesiologist was responsible for conducting the delirium assessment during these time slots. Simultaneously, the visual analog scale (VAS) was used to measure pain levels on a scale from 0 to 10, with lower scores indicating less pain Postoperative delirium (POD) was identified through the Confusion Assessment Method (CAM) (17), while the Memorial Delirium Assessment Scale (MDAS) was employed to measure the severity of POD (18). Furthermore, cognitive capabilities were evaluated within 6 months using the modified Telephone Interview for Cognitive Status (TICS-m). To gauge the quality of life, the World Health Organization Quality of Life brief version (WHOQOLBREF) was utilized.

### Statistical analysis

A preliminary test revealed that four covariates were expected to be included in the Logistic regression model. Based on the assumption of a 20% rate of loss to follow-up and a POD incidence of 10%, the necessary sample size was calculated to be 500 participants. This sample size was determined to ensure sufficient statistical power and validity for the study's objectives. In this study, we tested normality using the Kolmogorov–Smirnov test. For measurement data following a normal distribution, it was represented as mean ± standard deviation (SD). On the other hand, for measurement data exhibiting a skewed distribution, we used the median (p25, p75) or a number (%) to describe the data. To compare the baseline characteristics between the POD group and the NPOD group, we employed the chi-square test for categorical variables while the Mann–Whitney *U*-test was used for continuous variables. These statistical tests were chosen based on the nature of the data and are suitable for analyzing the differences between the two groups effectively. Normality was assessed with the Kolmogorov–Smirnov test. Measurement data that conformed to a normal distribution were presented as mean ± standard deviation (SD); on the other hand, measurement data with a skewed distribution were expressed as the median (p25, p75) or in percentage form (%).

First, we analyzed the risk and protective factors for POD using the multivariate logistic regression. In order to eliminate the influence of confounding factors, we conducted multiple sensitivity analyses in this study and three correction models were constructed: (1) age (50–90 years), sex, years of education and MMSE scores were included as covariates for correction; (2) age (50–90 years), sex, years of education, MMSE scores, as well as the history of smoking, drinking, hypertension and diabetes were included as covariates for correction; and (3) age ≥65 years, sex, years of education, MMSE scores, as well as the history of smoking, drinking, hypertension and diabetes were included as covariates for correction.

Second, assess the predictive capabilities of serum prealbumin or serum albumin and the combination of serum prealbumin + serum albumin + CSF POD biomarkers for POD, we utilized the receiver operating characteristic (ROC) curve and the precision-recall curve (PRC). Additionally, we employed Calibration to validate the prediction model and developed a nomogram that incorporated these independent variables for further analysis.

Third, we sought to explore whether the relationship between serum prealbumin or serum albumin and POD was influenced by CSF POD biomarkers by means of Stata MP16.0 (Solvusoft Corporation, Inc, Chicago, Illinois, USA). We utilized logistic regression models based on specific methods for this analysis. The first equation involved regressing the mediator variable (CSF POD biomarkers) on the independent variable (serum prealbumin or serum albumin). Subsequently, in the second equation, we regressed the dependent variable (POD) on the independent variable (serum prealbumin or serum albumin). Finally, in the third equation, we regressed the dependent variable on both the independent variable and the mediator variable. To estimate the attenuation or indirect effect, we conducted 10,000 bootstrapped iterations while controlling for age, years of education, gender, and MMSE in each path of the model.

Finally, we conducted an internal validation study to verify the predictive accuracy of the combined use of serum prealbumin and serum albumin + CSF POD biomarkers for POD. The model was visualized using ROC curve and clinical decision curve analysis (DCA). DynNom and Shiny packages were exploited to generate an online calculator (https://www.shinyapps.io/). Ten patients with POD occurring from February 2023 to October 2023 were selected for internal validation.

SPSS statistical software, version 25.0 (SPSS, Inc. Chicago, IL, USA), R software version 4.3.1 (R Foundation for Statistical Computing, Vienna, Austria), Stata MP16.0 (Solvusoft Corporation, Inc, Chicago, Illinois, USA) and GraphPad Prism version 7.00 (GraphPad Software, San Diego, CA) were used for statistical analyses and figure preparation. *P*-value < 0.05 was considered significant, except where specifically noted.

## Results

### Intergroup comparisons

**Figure 2:**
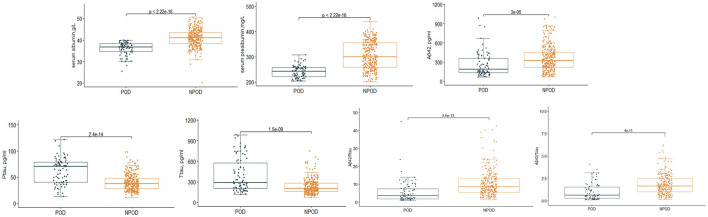
Distribution of serum prealbumin and serum albumin and biomarker levels for participants with and without delirium during subsequent hospitalization.

We observed a POD incidence of 23.1% (*n* = 84 of the 364 patients) The POD group and NPOD group displayed significant differences (*P* < 0.05) in serum prealbumin and serum albumin and various CSF biomarkers, including Aβ_42_, T-tau, P-tau, Aβ_42_/T-tau, and Aβ_42_/P-tau, as shown in Figure 2. Interestingly, there were no statistically significant variations in the preoperative MMSE score and the cognitive function at the 6-month postoperative follow-up visit between the two groups. A comprehensive overview of the participants' demographic and clinical data can be found in Table 1.

**Table 1:** Characteristics of participants in PNDABLE.

**Characteristic**	**NPOD (*n* = 280)**	**POD (*n* = 84)**	***P*-value**
Age [year, *M* (*Q*)]	60.00 (12)	74.00 (8)^*^	< 0.001
Male [*n* (%)]	169 (60.36)	47 (55.95)	0.471
BMI [kg.m^−2^, *M* (*Q*)]	25.39 (4.8)	25.52 (3.77)	0.855
Education [year, *M* (*Q*)]	9 (3)	9 (5)	0.253
MMSE [scores, *M* (*Q*)]	28.00 (3)	28.00 (1)	0.148
Smoking history [*n* (%)]	85 (30.36)	18 (21.43)	0.111
Drinking history [*n* (%)]	98 (35)	19 (22.62)^*^	0.033
Hypertension [*n* (%)]	94 (33.57)	41 (48.81)^*^	0.011
Diabetes [*n* (%)]	48 (17.14)	22 (26.19)	0.065
Serum albumin [g/L, *M* (*Q*)]	41.08 (5.28)	36.77 (3.85)^*^	< 0.001
Serum prealbumin [mg/L, *M* (*Q*)]	299.5 (98.5)	242.0 (36.5)^*^	< 0.001
MDAS [scores, *M* (*Q*)]	1.5 (6)	13.0 (3)^*^	< 0.001
Aβ42 [pg/ml, *M* (*Q*)]	323.04 (233.60)	187.66 (238.87)^*^	< 0.001
P-tau [pg/ml, *M* (*Q*)]	37.55 (18.92)	70.33 (38.14)^*^	< 0.001
T-tau [pg/ml, *M* (*Q*)]	202.01 (122.90)	288.76 (392.04)^*^	< 0.001
Aβ_42_/P-tau [*M* (*Q*)]	8.46 (7.50)	3.55 (5.74)^*^	< 0.001
Aβ_42_/T-tau [*M* (*Q*)]	1.64 (1.49)	0.62 (1.31)^*^	< 0.001
Duration of anesthesia [min, *M* (*Q*)]	140.00 (30)	140.00 (30)	0.252
Duration of surgery [min, *M* (*Q*)]	120.00 (20)	120.00 (20)	0.723
Estimated volume of infusion [ml, *M* (*Q*)]	800.00 (100)	900.00 (100)	0.140
Estimated blood loss [ml, *M* (*Q*)]	120.00 (20)	120.00 (23.75)	0.543
TICS-m score [scores, *M* (*Q*)]	36.00 (5)	36.00 (4)	0.438
**WHOQOL-BREF score**			
Physical domain [scores, *M* (*Q*)]	69.00 (5)	69 (5.75)	0.165
Psychological domain [scores, *M* (*Q*)]	76.00 (4)	75.00 (6)	0.410
Social relationships domain [scores, *M* (*Q*)]	68.00 (3)	68.00 (4)	0.824
Environment domain [scores, *M* (*Q*)]	84.00 (4)	84.00 (4)	0.437

Continuous variable use Student's t test or Mann–Whitney U, Categorical variable use Chi-square test.

The numerical variables of normal distribution are statistically described by average ± standard deviation [$\bar{x}$ ± s].

Non-normally distributed numerical variables are statistically described by Median (Interquartile spacing) [M (Q)].

Categorical variables are statistically described by Sample size (Percent) [n (%)].

^*^P < 0.05.

### Logistic regression analysis of the influencing factors of POD

CSF levels of T-tau and P-tau emerged as significant risk factors for POD. Conversely, the univariate analysis revealed that serum prealbumin and serum albumin and CSF levels of Aβ_42_, Aβ_42_/T-tau, and Aβ_42_/P-tau were protective factors against POD. After adjusting for potential confounding variables such as age, years of education, gender, MMSE score, as well as history of diabetes, smoking, drinking, and hypertension, the multivariate logistic regression assessment maintained the consistency of these results. Furthermore, sensitivity analyses were conducted, and the outcomes corroborated the findings, as shown in Table 2.

**Table 2:** Logistic regression on analysis and sensitivity analysis in PNDABLE study.

	**Model 1** ^**a**^		**Model 2** ^**b**^		**Model 3** ^**c**^		**Model 4** ^**d**^	
	**OR (95% CI)**	***P*** **-value**	**OR (95% CI)**	***P*** **-value**	**OR (95% CI)**	***P*** **-value**	**OR (95% CI)**	***P*** **-value**
Serum albumin, g/L	0.690 (0.626–0.759)	0.000^*^	0.712 (0.623–0.814)	0.000^*^	0.702 (0.612–0.805)	0.000^*^	0.665 (0.567–0.779)	0.000^*^
Serum prealbumin, mg/L	0.970 (0.962–0.978)	0.000^*^	0.959 (0.945–0.973)	0.000^*^	0.956 (0.941–0.971)	0.000^*^	0.953 (0.936–0.970)	0.000^*^
Aβ_42_, pg/ml	0.997 (0.995–0.998)	0.000^*^	0.997 (0.996–0.999)	0.000^*^	0.997 (0.995–0.999)	0.013^*^	0.997 (0.995–0.999)	0.012
P-tau, pg/ml	1.059 (1.044–1.075)	0.000^*^	1.068 (1.043–1.093)	0.000^*^	1.069 (1.044–1.094)	0.000^*^	1.075 (1.047–1.104)	0.000^*^
T-tau, pg/ml	1.006 (1.004–1.007)	0.000^*^	1.006 (1.003–1.009)	0.000^*^	1.006 (1.004–1.009)	0.000^*^	1.006 (1.003–1.009)	0.000^*^
Aβ_42_/P-tau	0.840 (0.787–0.897)	0.000^*^	0.857 (0.797–0.922)	0.000^*^	0.853 (0.793–0.918)	0.000^*^	0.854 (0.793–0.921)	0.000^*^
Aβ_42_/T-tau	0.395 (0.284–0.550)	0.000^*^	0.473 (0.315–0.710)	0.000^*^	0.459 (0.303–0.697)	0.000^*^	0.462 (0.304–0.703)	0.000^*^

OR, odds ratio; 95%CI, 95% confidence interval; Aβ_42_, β-amyloid42; T-tau, total tau; P-tau, phosphorylated tau.

^a^Model 1: unadjusted.

^b^Model 2: adjusted for age (50–90), sex, years of education and MMSE score.

^c^Model 3: adjusted for age (50–90), sex, years of education, MMSE scores, smoking history, drinking history, hypertension and diabetes.

^d^Model 4: adjusted for age ≥65, sex, years of education, MMSE scores, smoking history, drinking history, hypertension and diabetes.

^*^P < 0.05.

### Causal mediation analyses

**Figure 3:**
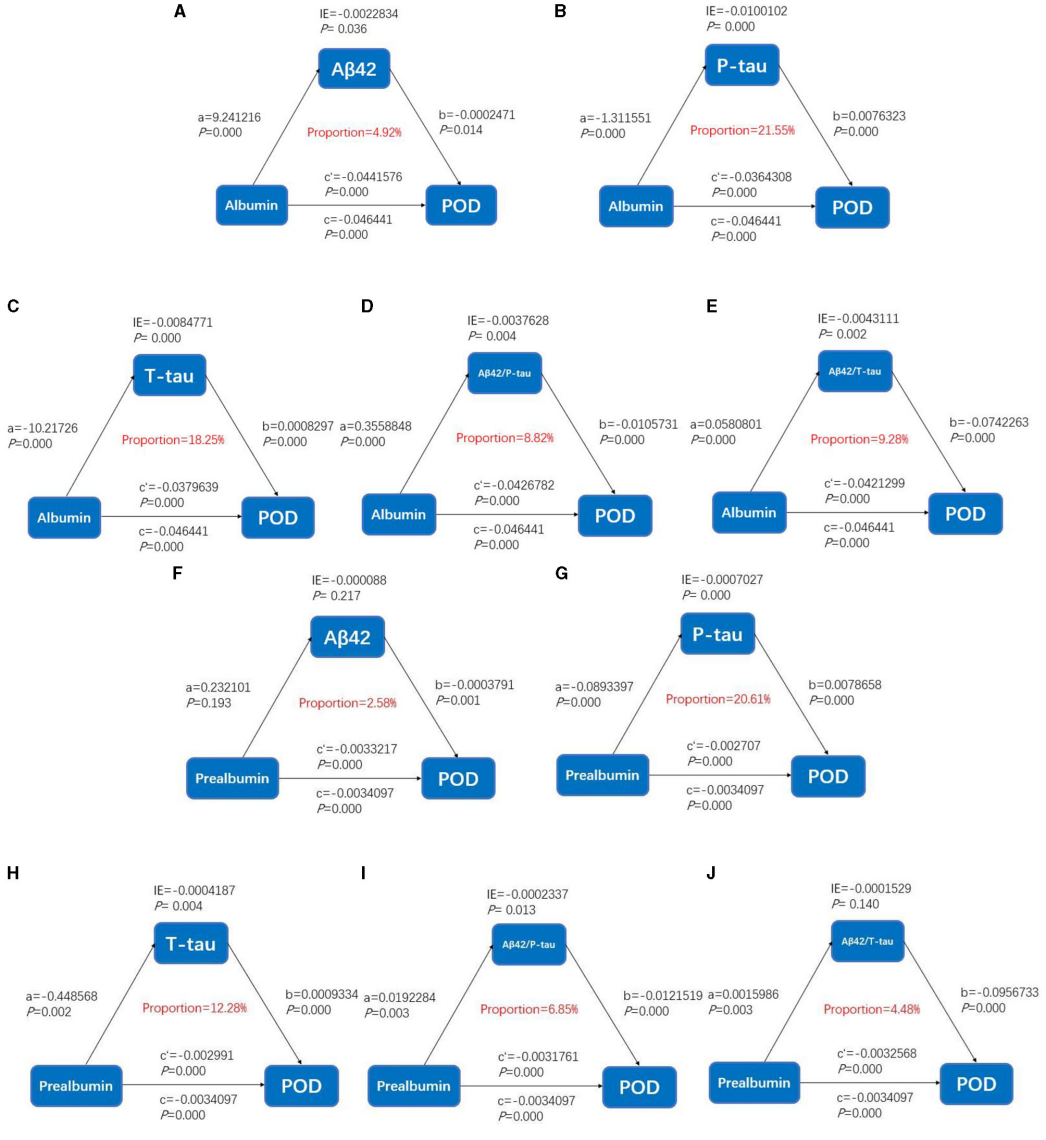
Mediation analyses with postoperative delirium as the cognitive outcome. In the PNDABLE (Perioperative Neurocognitive Disorder And Biomarker LifestylE study), the relationship between serum prealbumin and serum albumin and postoperative delirium was mediated by amyloid pathology indicated by **(A, F)** amyloid β 42 (Aβ_42_), **(B, G)** phosphorylated total-tau (P-tau), **(C, H)** Total-tau (T-tau), **(D, I)** amyloid β 42/phosphorylated total-tau (Aβ_42_/P-tau), and **(E, J)** amyloid β 42/Total-tau (Aβ_42_/T-tau). IE, indirect effect.

**Figure 4:**
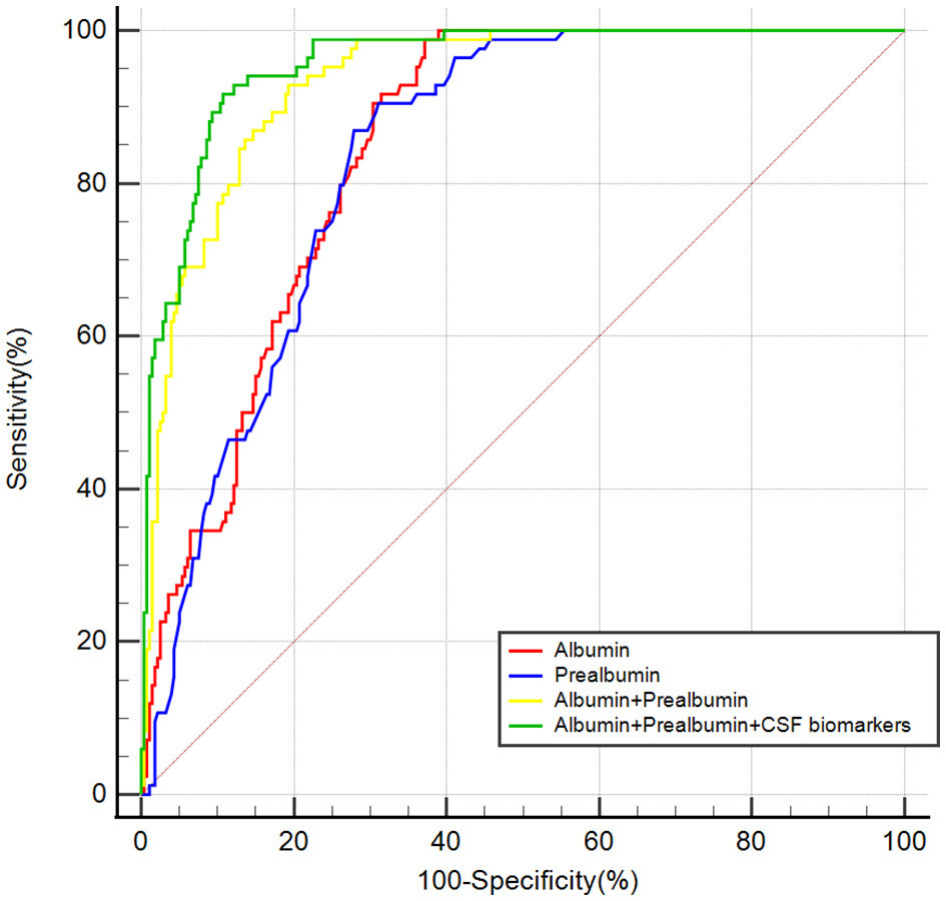
The ROC curve analysis of serum prealbumin, serum albumin, the combination of serum prealbumin and serum albumin and the combination of serum prealbumin and serum albumin and POD biomarkers showed that the combination of serum prealbumin and serum albumin and POD biomarkers had a high diagnostic value for POD.

As shown in Figure 4, the relationship between serum prealbumin and POD was found to be partially mediated by T-tau and P-tau, with their mediation proportions of 12.28 and 20.61%, respectively. The relationship between serum albumin and POD was found to be partially mediated by T-tau and P-tau, with their mediation proportions of 18.28 and 21.55%, respectively (Figure 3).

### A predictive model and an internal validation study for POD

**Figure 5:**
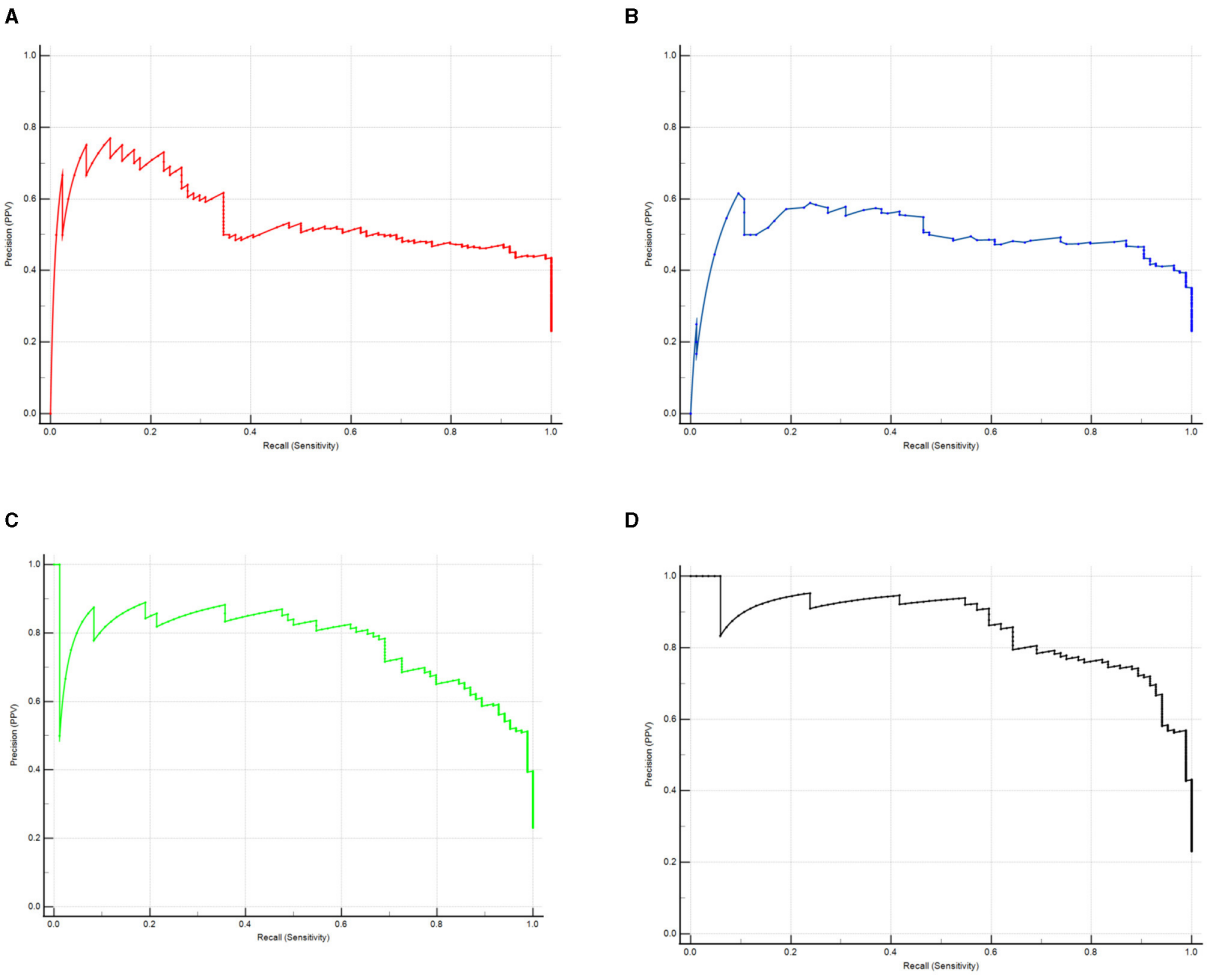
The PRC curve analysis of serum prealbumin **(A)**, serum albumin **(B)**, the combination of serum prealbumin and serum albumin **(C)** and the combination of serum prealbumin and serum albumin and POD biomarkers **(D)** showed that the combination of serum prealbumin and serum albumin and POD biomarkers had a high diagnostic value for POD.

ROC curve showed that the model combining serum prealbumin and serum albumin and POD biomarkers (AUC = 0.957; *P* < 0.001) exhibited a better discriminatory ability to predict POD compared to serum prealbumin alone (AUC = 0.836; *P* < 0.001), serum albumin alone (AUC = 0.848; *P* < 0.001) and the combination of serum prealbumin and serum albumin (AUC = 0.934; *P* < 0.001) (Figure 4). PRC curve showed that the combination of serum prealbumin and serum albumin and POD biomarkers (AUC = 0.854) exhibited a better discriminatory ability to predict POD compared to serum prealbumin alone (AUC = 0.496), serum albumin alone (AUC = 0.547) and the combination of serum prealbumin and serum albumin (AUC = 0.770; Figure 5).

**Figure 6:**
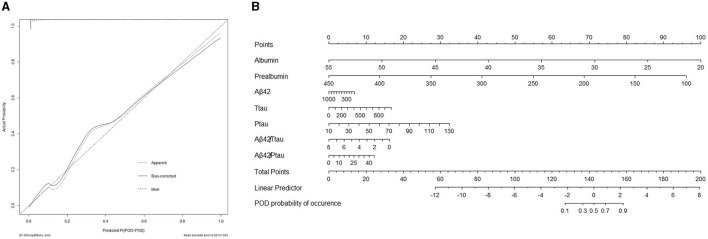
**(A)** Calibration curve presented prediction of POD after surgery between the prediction model and actual observation. The Hosmer-Lemeshow test indicated a good prediction of the nomogram. **(B)** Nomogram for predicting POD after surgery.

**Figure 7:**
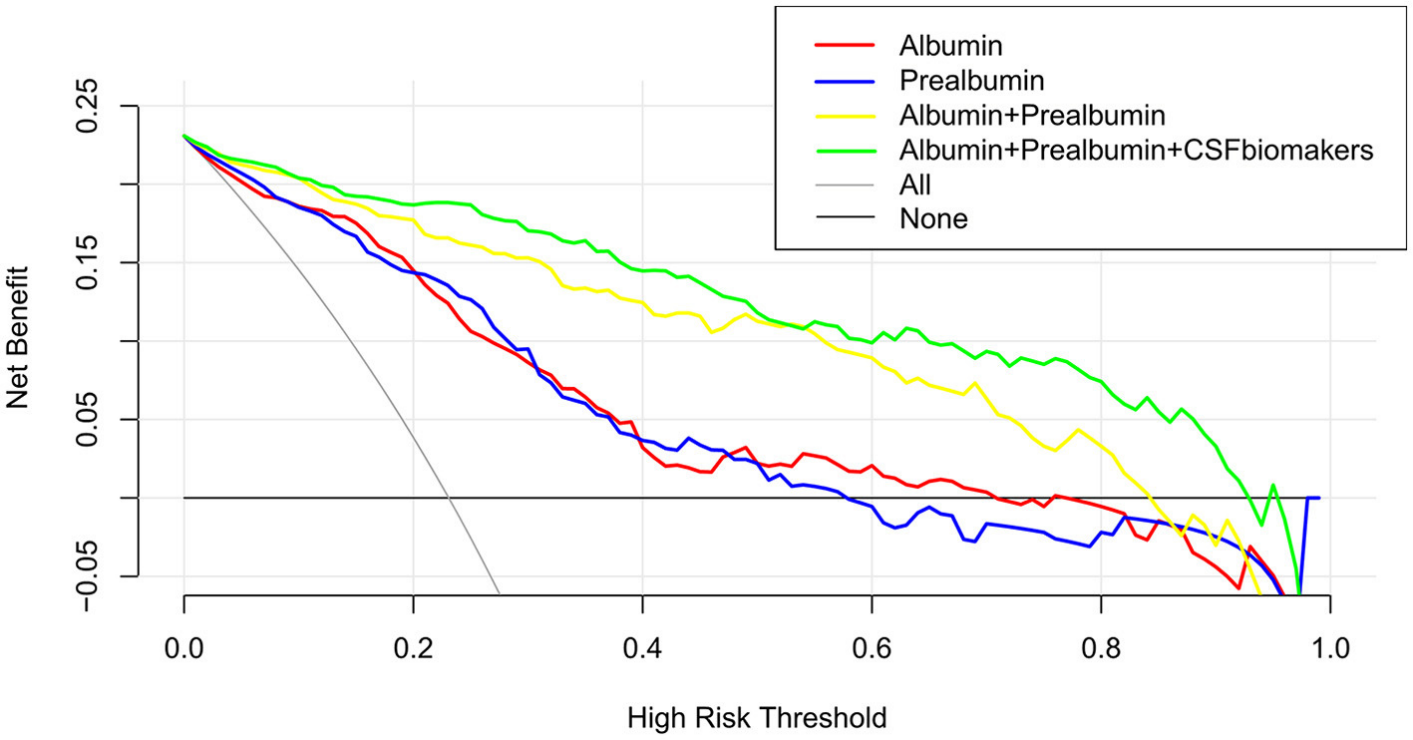
The clinical DCA was plotted using R software, and the results showed that the model of the combination of serum prealbumin and serum albumin and POD biomarkers brought a high benefit value to patients.

**Figure 8:**
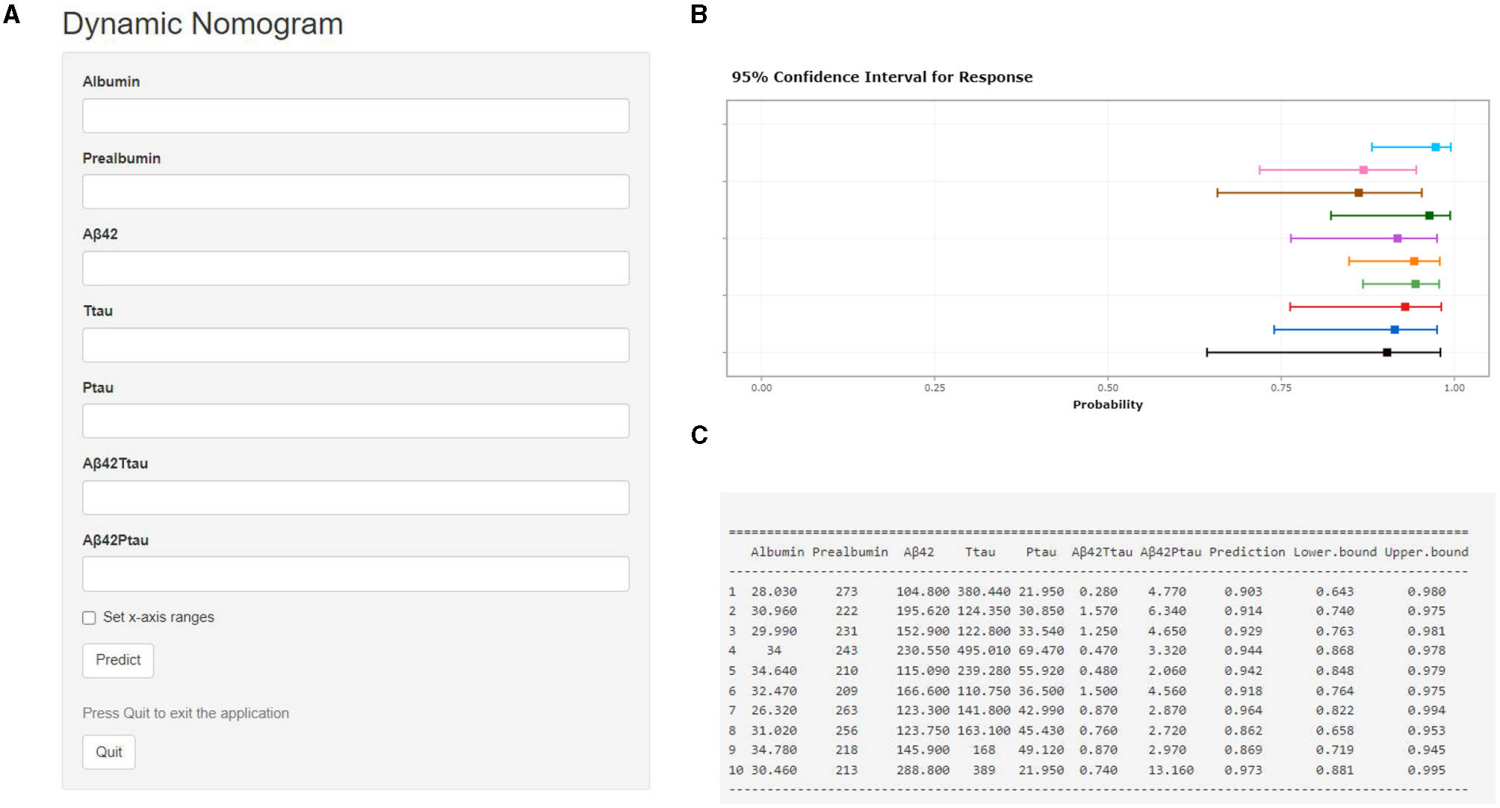
Visualization for predictive model. The online calculator translated from the nomogram for generating risk of POD by the internal verification. Users can submit values for the seven features into the corresponding text box of the web page through the computer or mobile phone for calculation **(A)**. Once the output of the sample has been calculated, the results page will display the probability of POD, the 95% confidence interval, and the parameters of the model **(B, C)**.

A calibration plot comparing the prediction of POD between the model and actual observation was created with the result of the Hosmer–Lemeshow test (*P* = 0.563), indicating good predictive accuracy (Figure 6A). We established a nomogram incorporating these independent variables (Figure 6B). In addition, to assess the clinical usefulness of the nomogram, the R software was used to plot the clinical DCA. The DCA provided insight into the range of predicted risks, and the results showed that the model delivered a high predictive value for POD patients (Figure 7) and then translated it to a dynamic online calculator (https://byl-qdsslyy.shinyapps.io/dynnomapp-1) aiming at promoting the clinical utility of our findings. The dynamic online calculator can accurately predict the occurrence of POD by selecting POD patients for the internal validation study (Figure 8).

## Discussion

In the present study, the incidence of POD is 23.1%, which is consistent with the POD incidence range of 3.6%−41% reported in a previous study (19). Serum prealbumin, serum albumin and CSF levels of Aβ_42_, T-tau, P-tau, Aβ_42_/T-tau, and Aβ_42_/P-tau were significantly different between the POD group and NPOD group, but there were no statistically significant variations in the preoperative MMSE score and the cognitive function at the follow-up visit between the two groups. In addition, we found that lower serum prealbumin and serum albumin increased the incidence of POD, indicating that malnutrition was associated with an increased risk of POD, but there was no cognitive impairment before surgery and postoperative 6-month.

Aβ plays an important role in the progression of neurological diseases such as Alzheimer's disease. To be specific, the aggregation and deposition of Aβ are believed to be closely related to the development and deterioration of AD (20). Townsend et al. injected Aβ in the condensed state into the cerebral cortex of rats and monkeys, and electron microscopy showed that cortical tissue necrosis, gliosis, and peripheral neuronal loss after injection had significant and positive dose-dependent associations with the injected Aβ (21). Increased Aβ levels in the hippocampus after anesthesia are considered to be one of the possible mechanisms of POCD. Recently, it has been reported that increased Aβ levels in brain have a causal relationship with cognitive dysfunction (22). Other studies have found that direct injections of condensed Aβ into the hippocampus of rats can lead to decreased learning and memory (23). Tau protein is a low molecular weight glycoprotein abundantly found in central nervous cells. Tau protein in normal state has a stabilizing effect on microtubules. When the phosphorylation, glycosylation and ubiquitination of tau proteins are abnormal, the physiological function of nerve fibers is impaired (24). Studies have shown that Aβ production and tau phosphorylation contribute to the development of AD (25). Intracranial injection of agglutinated Aβ led to increased tau phosphorylation at specific sites and neuronal apoptosis. The hippocampus is the most important brain region for learning and memory, particularly short-term memory (26). Accumulated Aβ has severe neurotoxic properties, and the hippocampus is the most vulnerable target organ (27). It has been confirmed that the hippocampal injection of Aβ can significantly reduce the learning and memory abilities of rats, and the receptor for advanced glycation end products (RAGE) in microglia may partially mediate the neurotoxicity of Aβ. Aβ can bind to RAGE, and directly or indirectly activate microglia to release a large number of cytotoxic substances, such as inflammatory mediators and free, inducing cell apoptosis (28). Increased production, abnormal metabolism, and impaired transport of Aβ can lead to its accumulation in the brain, resulting in severe neurotoxicity.

Oxidative stress refers to some physiological processes in the body, such as tissue damage, hypoxia, serious diseases and infections, which may lead to increased oxygen consumption or decreased oxygen supply and even brain diseases. Some studies have found that oxidative stress or antioxidant deficiencies can increase damage to brain tissue, leading to cognitive decline (29). Pathological injuries such as hypoxia and ischemia-reperfusion may lead to decreased brain oxidative metabolism as well as associated cognitive and behavioral symptoms. A previous study found that (30) there were no significant differences in plasma oxidative stress biomarkers [superoxide dismutase (SOD) and malondialdehyde (MDA)] between POD group and control group before surgery, while the plasma SOD and MDA levels in POD group increased significantly after operation, suggesting that excessive oxidative stress might be involved in POD occurrence. Another previous study showed that serum levels of oxidative stress biomarkers were significantly elevated in POD group after operation (31). This study also confirmed that oxidative stress plays an important role in the pathogenesis and treatment of POD and proposed that oxidative stress depressants could relieve POD symptoms. Neuroinflammation is a major source of free radicals, reactive oxygen species (ROS) and reactive nitrogen species (RNS) produced by the immune response of the central nervous system (CNS), which could alter proteins, lipids and nucleic acids. Neurons are highly susceptible to the harmful effects of these active substances. The overproduction of active species in biological systems and the lack of enzymatic and non-enzymatic antioxidants can lead to oxidative stress. ROS accumulation and oxidative damage are two markers of cellular aging. Apart from being the main source of ROS, mitochondria are also the main target of oxidative damage, which in turn reduces mitochondrial efficiency and leads to the production of more ROS, forming a vicious cycle (32). Decreased tetrahydrobiotrexate, impaired serotonergic neurotransmission and increased oxidative stress have also been found to be closely related to POD pathogenesis (33).

In our study, we found that the association between serum prealbumin and serum albumin and POD was mediated by T-tau and P-tau. When the level of prealbumin and albumin decreases, the generation of oxygen free radicals increases and the oxidative defense ability decreases, leading to oxidative stress reaction (34). Oxidative stress is caused by the imbalance of aerobic metabolism, and it poses a serious threat to cellular homeostasis. Highly reactive oxygen species oxidize lipids, proteins and DNA, leading to tissue damage and cell death. Previous studies have found that oxidative stress is associated with a decrement in the expression of brain-derived neurotrophic factor (BDNF), which plays an important role in the consolidation of new memories. In addition, BDNF secreted by activated microglia cells can also exert neuroprotective effects (35). Other studies have found that the low expression of BDNF and its receptor trkBr can up-regulate the expression of GSK-3b by inhibiting the activation of PI3K/AKT pathway, and ultimately lead to tau hyperphosphorylation (36). Abnormally hyperphosphorylated tau loses its biological function of promoting microtubule assembly and maintaining microtubule stability (37). P-tau competes with tubulin in binding to normal tau and thus destructs the normal microtubule system, which inhibits the formation and function of synapses (38, 39), impairs learning and memory, and eventually leads to the occurrence of POD.

From the ROC and PRC analyses, we can see that the model combining serum albumin and POD biomarkers can be used to predict the occurrence of POD. A calibration plot comparing the prediction of POD between the model and actual observation was created with the result of the Hosmer–Lemeshow test, showing good predictive accuracy. The DCA provided insight into the range of predicted risks, showing that the model was of high predictive value for POD patients. The dynamic online calculator can accurately predict the occurrence of POD by selecting POD patients for the internal validation study. Therefore, our results provide more precise and personalized risk prediction by the DCA and the internal verification.

Our study also has three limitations. Firstly, since the PNDABLE study is an ongoing prospective cohort, we need to continuously update the database during the follow-up in the future. Secondly, since this study is a single-center study, our findings need to be further verified by multi-center studies. Thirdly, this study only focused on the relationship between serum prealbumin or serum albumin and POD, without taking other factors related to POD pathogenesis into consideration, which might result in confounding.

## Conclusion

Preoperative low serum prealbumin and serum albumin levels were associated with the occurrence of POD, which was partly mediated by CSF T-tau and P-tau. Our internal verification study showed that the model combining serum prealbumin, serum albumin and CSF POD biomarkers could accurately predict the occurrence of POD.

## Data availability statement

The original contributions presented in the study are included in the article/supplementary material, further inquiries can be directed to the corresponding author.

## Ethics statement

The studies involving humans were approved by Ethics Committee of Qingdao Municipal Hospital. The studies were conducted in accordance with the local legislation and institutional requirements. The participants provided their written informed consent to participate in this study.

## Author contributions

BW: Data curation, Formal analysis, Funding acquisition, Investigation, Methodology, Project administration, Writing – original draft. YX: Data curation, Formal analysis, Writing – original draft. XT: Data curation, Formal analysis, Investigation, Writing – original draft. FW: Data curation, Formal analysis, Writing – original draft. SH: Investigation, Writing – original draft. YY: Investigation, Writing – original draft. SX: Investigation, Writing – original draft. HG: Investigation, Writing – original draft. RD: Investigation, Writing – original draft. YL: Data curation, Writing – original draft. CL: Data curation, Writing – original draft. XL: Investigation, Writing – original draft. YB: Funding acquisition, Methodology, Project administration, Supervision, Writing – original draft, Writing – review & editing.

## References

[1] Evered L Silbert B Knopman DS Scott DA Dekosky ST Rasmussen LS. Recommendations for the nomenclature of cognitive change associated with anaesthesia and surgery-2018. Br J Anaesth. (2018) 121:1005–12. 10.1097/ALN.0000000000002334 30336844 PMC7069032

[2] Safavynia S Goldstein P. The role of neuroinflammation in postoperative cognitive dysfunction: moving from hypothesis to treatment. Front Psychiatry. (2018) 9:752. 10.3389/fpsyt.2018.00752 30705643 PMC6345198

[3] Steinmetz J Christensen K Lund T Lohse N Rasmussen L. Long-term consequences of postoperative cognitive dysfunction. Anesthesiology. (2009) 110:548–55. 10.1097/ALN.0b013e318195b569 19225398

[4] Ramaiah R Lam A. Postoperative cognitive dysfunction in the elderly. Anesthesiol Clin. (2009) 27:485–96. 10.1016/j.anclin.2009.07.011 19825488

[5] Guo T Noble W Hanger D. Roles of tau protein in health and disease. Acta Neuropathol. (2017) 133:665–704. 10.1007/s00401-017-1707-9 28386764 PMC5390006

[6] Fong T Vasunilashorn S Gou Y Libermann T Dillon S Schmitt E. Association of CSF Alzheimer's disease biomarkers with postoperative delirium in older adults. Alzheimers Dement. (2021) 7:e12125. 10.1002/trc2.12125 33748398 PMC7968120

[7] Cunningham EL Mcguinness B Mcauley DF Toombs J Mawhinney T O'brien S. CSF beta-amyloid 1-42 concentration predicts delirium following elective arthroplasty surgery in an observational cohort study. Ann Surg. (2019) 269:1200–5. 10.1097/SLA.0000000000002684 31082921

[8] Dutkiewicz R Zetterberg H Andreasson U Blennow K Nellgård B. Dementia and CSF-biomarkers for Alzheimer's disease predict mortality after acute hip fracture. Acta Anaesthesiol Scand. (2020) 64:93–103. 10.1111/aas.13472 31508810

[9] Ingenbleek Y Young VR. Significance of transthyretin in protein metabolism. Clin Chem Lab Med. (2002) 40:1281–91. 10.1515/CCLM.2002.222 12553432

[10] Hornok V Amin KWK Kovács AN Juhász Á Katona G Balogh GT. Increased blood-brain barrier permeability of neuroprotective drug by colloidal serum albumin carriers. Colloids Surf B Biointerfaces. (2022) 220:112935. 10.1016/j.colsurfb.2022.112935 36265318

[11] Vita GM De Simone G De Marinis E Nervi C Ascenzi P Di Masi A. Serum albumin and nucleic acids biodistribution: from molecular aspects to biotechnological applications. IUBMB Life. (2022) 74:866–79. 10.1002/iub.2653 35580148

[12] Leung J Leung V Leung CM Pan PC. Clinical utility and validation of two instruments (the Confusion Assessment Method Algorithm and the Chinese version of Nursing Delirium Screening Scale) to detect delirium in geriatric inpatients. Gen Hosp Psychiatry. (2008) 30:171–6. 10.1016/j.genhosppsych.2007.12.007 18291299

[13] Montagne A Barnes SR Sweeney MD Halliday MR Sagare AP Zhao Z. Blood-brain barrier breakdown in the aging human hippocampus. Neuron. (2015) 85:296–302. 10.1016/j.neuron.2014.12.032 25611508 PMC4350773

[14] Sweeney MD Sagare AP Zlokovic BV. Cerebrospinal fluid biomarkers of neurovascular dysfunction in mild dementia and Alzheimer's disease. J Cereb Blood Flow Metab. (2015) 35:1055–68. 10.1038/jcbfm.2015.76 25899298 PMC4640280

[15] Cortese GP Burger C. Neuroinflammatory challenges compromise neuronal function in the aging brain: postoperative cognitive delirium and Alzheimer's disease. Behav Brain Res. (2017) 322:269–79. 10.1016/j.bbr.2016.08.027 27544872 PMC5450823

[16] Geng J Zhang Y Chen H Shi H Wu Z Chen J. Associations between Alzheimer's disease biomarkers and postoperative delirium or cognitive dysfunction: a meta-analysis and trial sequential analysis of prospective clinical trials. Eur J Anaesthesiol. (2024) 41:234–44. 10.1097/EJA.0000000000001933 38038408 PMC10842675

[17] Chung D Sue A Hughes S Simmons J Hailu T Swift C. Impact of race/ethnicity on pain management outcomes in a community-based teaching hospital following inpatient palliative care consultation. Cureus. (2016) 8:e823. 10.7759/cureus.823 27882270 PMC5106348

[18] Inouye SK Van Dyck CH Alessi CA Balkin S Siegal AP Horwitz RI. Clarifying confusion: the confusion assessment method. A new method for detection of delirium. Ann Intern Med. (1990) 113:941–8. 10.7326/0003-4819-113-12-941 2240918

[19] Leung J Sands L Lim E Tsai T Kinjo S. Does preoperative risk for delirium moderate the effects of postoperative pain and opiate use on postoperative delirium? Am J Geriatr Psychiatry. (2013) 21:946–56. 10.1016/j.jagp.2013.01.069 23659900 PMC3742555

[20] Kowall NW Beal MF Busciglio J Duffy LK Yankner BA. An *in vivo* model for the neurodegenerative effects of beta amyloid and protection by substance P. Proc Natl Acad Sci U S A. (1991) 88:7247–51. 10.1073/pnas.88.16.7247 1714596 PMC52271

[21] Townsend KP Obregon D Quadros A Patel N Volmar C Paris D. Proinflammatory and vasoactive effects of Abeta in the cerebrovasculature. Ann N Y Acad Sci. (2002) 977:65–76. 10.1111/j.1749-6632.2002.tb04799.x 12480734

[22] Sharma C Kim S Nam Y Jung UJ Kim SR. Mitochondrial dysfunction as a driver of cognitive impairment in Alzheimer's disease. Int J Mol Sci. (2021) 22. 10.3390/ijms22094850 34063708 PMC8125007

[23] Qian C Yang C Lu M Bao J Shen H Deng B. Activating AhR alleviates cognitive deficits of Alzheimer's disease model mice by upregulating endogenous Aβ catabolic enzyme Neprilysin. Theranostics. (2021) 11:8797–812. 10.7150/thno.61601 34522212 PMC8419060

[24] Yatin SM Aksenova M Aksenov M Markesbery WR Aulick T Butterfield DA. Temporal relations among amyloid beta-peptide-induced free-radical oxidative stress, neuronal toxicity, and neuronal defensive responses. J Mol Neurosci. (1998) 11:183–97. 10.1385/JMN:11:3:183 10344789

[25] Ramezani M Darbandi N Khodagholi F Hashemi A. Myricetin protects hippocampal CA3 pyramidal neurons and improves learning and memory impairments in rats with Alzheimer's disease. Neural Regen Res. (2016) 11:1976–80. 10.4103/1673-5374.197141 28197195 PMC5270437

[26] Martin SB Dowling AL Head E. Therapeutic interventions targeting beta amyloid pathogenesis in an aging dog model. Curr Neuropharmacol. (2011) 9:651–61. 10.2174/157015911798376217 22654723 PMC3263459

[27] Deane R Du Yan S Submamaryan RK Larue B Jovanovic S Hogg E. RAGE mediates amyloid-beta peptide transport across the blood-brain barrier and accumulation in brain. Nat Med. (2003) 9:907–13. 10.1038/nm890 12808450

[28] Maldonado JR. Neuropathogenesis of delirium: review of current etiologic theories and common pathways. Am J Geriatr Psychiatry. (2013) 21:1190–222. 10.1016/j.jagp.2013.09.005 24206937

[29] Netto MB De Oliveira Junior AN Goldim M Mathias K Fileti ME Da Rosa N. Oxidative stress and mitochondrial dysfunction contributes to postoperative cognitive dysfunction in elderly rats. Brain Behav Immun. (2018) 73:661–9. 10.1016/j.bbi.2018.07.016 30041011

[30] Karlidag R Unal S Sezer OH Bay Karabulut A Battaloglu B But A. The role of oxidative stress in postoperative delirium. Gen Hosp Psychiatry. (2006) 28:418–23. 10.1016/j.genhosppsych.2006.06.002 16950378

[31] Zhang J Gao J Guo G Li S Zhan G Xie Z. Anesthesia and surgery induce delirium-like behavior in susceptible mice: the role of oxidative stress. Am J Transl Res. (2018) 10:2435–44. 30210682 PMC6129548

[32] Egberts A Fekkes D Wijnbeld EH Van Der Ploeg MA Van Saase JL Ziere G. Disturbed serotonergic neurotransmission and oxidative stress in elderly patients with delirium. Dement Geriatr Cogn Dis Extra. (2015) 5:450–8. 10.1159/000440696 26955379 PMC4777943

[33] Roche M Rondeau P Singh NR Tarnus E Bourdon E. The antioxidant properties of serum albumin. FEBS Lett. (2008) 582:1783–7. 10.1016/j.febslet.2008.04.057 18474236

[34] Allen SJ Watson JJ Shoemark DK Barua NU Patel NK. GDNF, NGF and BDNF as therapeutic options for neurodegeneration. Pharmacol Ther. (2013) 138:155–75. 10.1016/j.pharmthera.2013.01.004 23348013

[35] Huang YN Lin CI Liao H Liu CY Chen YH Chiu WC. Cholesterol overload induces apoptosis in SH-SY5Y human neuroblastoma cells through the up regulation of flotillin-2 in the lipid raft and the activation of BDNF/Trkb signaling. Neuroscience. (2016) 328:201–9. 10.1016/j.neuroscience.2016.04.043 27155148

[36] John A Reddy PH. Synaptic basis of Alzheimer's disease: focus on synaptic amyloid beta, P-tau and mitochondria. Ageing Res Rev. (2021) 65:101208. 10.1016/j.arr.2020.101208 33157321 PMC7770124

[37] Didonna A. Tau at the interface between neurodegeneration and neuroinflammation. Genes Immun. (2020) 21:288–300. 10.1038/s41435-020-00113-5 33011744

[38] Li B Chohan MO Grundke-Iqbal I Iqbal K. Disruption of microtubule network by Alzheimer abnormally hyperphosphorylated tau. Acta Neuropathol. (2007) 113:501–11. 10.1007/s00401-007-0207-8 17372746 PMC3191942

[39] Roberson ED Scearce-Levie K Palop JJ Yan F Cheng IH Wu T. Reducing endogenous tau ameliorates amyloid beta-induced deficits in an Alzheimer's disease mouse model. Science. (2007) 316:750–4. 10.1126/science.1141736 17478722

